# Role of cardiac magnetic resonance in patients with chest pain and pulmonary aneurysm

**DOI:** 10.1186/1532-429X-13-S1-P388

**Published:** 2011-02-02

**Authors:** Paolo China, Manuel De Lazzari, Martina Perazzolo Marra, Barbara Bauce, Vittorio Pengo, Umberto Cucchini, Francesco Corbetti, Luisa Cacciavillani, Sabino Iliceto

**Affiliations:** 1 Department of Cardiac, Thoracic and Vascular Sciences, University of Padua, Padua, Italy; 2 Department of Radiology, Padua, Italy

## Introduction

Aneurysm of Main Pulmonary Artery (MPA) is a rare condition in which aetiology and pathogenesis are not well known. The diagnostic role of Cardiovascular Magnetic Resonance (CMR) in many pathological conditions is growing, because of its high spatial resolution and reproducibility. Moreover, the use of contrast agent allows the possibility to define tissue characterization even for vessel structures.

## Purpose

We reported two cases of MPA in which CMR had a fundamental role in achieving the diagnosis.

## Methods

We retrospectively evaluated the 250 patients hospitalized for acute chest pain with excluded coronary disease who underwent to CMR and in which MPA was shown. We collected two cases in which during the same hospitalization CMR was achieved by a 1.5-Tesla scanner (Magnetom Avanto, Siemens) using a comprehensive dedicated protocol. Clinical, echocardiographic and angiographic data were collected.

## Results

Case 1: 65-years-old woman with almost a year history of progressive exertional dyspnea and chest pain was referred to emergency department. Electrocardiogram did not show any abnormality both at rest and during stress. An echocardiogram revealed a dilation of MPA with a dysplastic pulmonary valve. Cardiac catheterization showed no coronary artery disease. CMR demonstrated a quadricuspid pulmonary valve with mild regurgitation accompanied by a giant aneurysm of the MPA, without signs of dissection. Case 2: 38-years-old woman affected by lupus eritematosus systemicus was admitted to our institution due to the recent onset of chest pain episodes that worsened with supine position. A CMR confirmed the MPA sever dilatation, and also revealed a diffuse delayed gadolinium enhancement of the MPA wall, particularly on the anterior portion. Pulmonary embolism and aortic dissection were excluded.

## Conclusions

In patients presenting with chest pain of unknown cause the presence of a MPA aneurysm, even if rare, should be consider in differential diagnosis with coronary artery disease, as usually happen for aortic aneurysm or dissection. Moreover, in our study CMR confirmed its capability to define the morphology and dimension of the MPAs, providing also additional information on tissue wall abnormalities.

